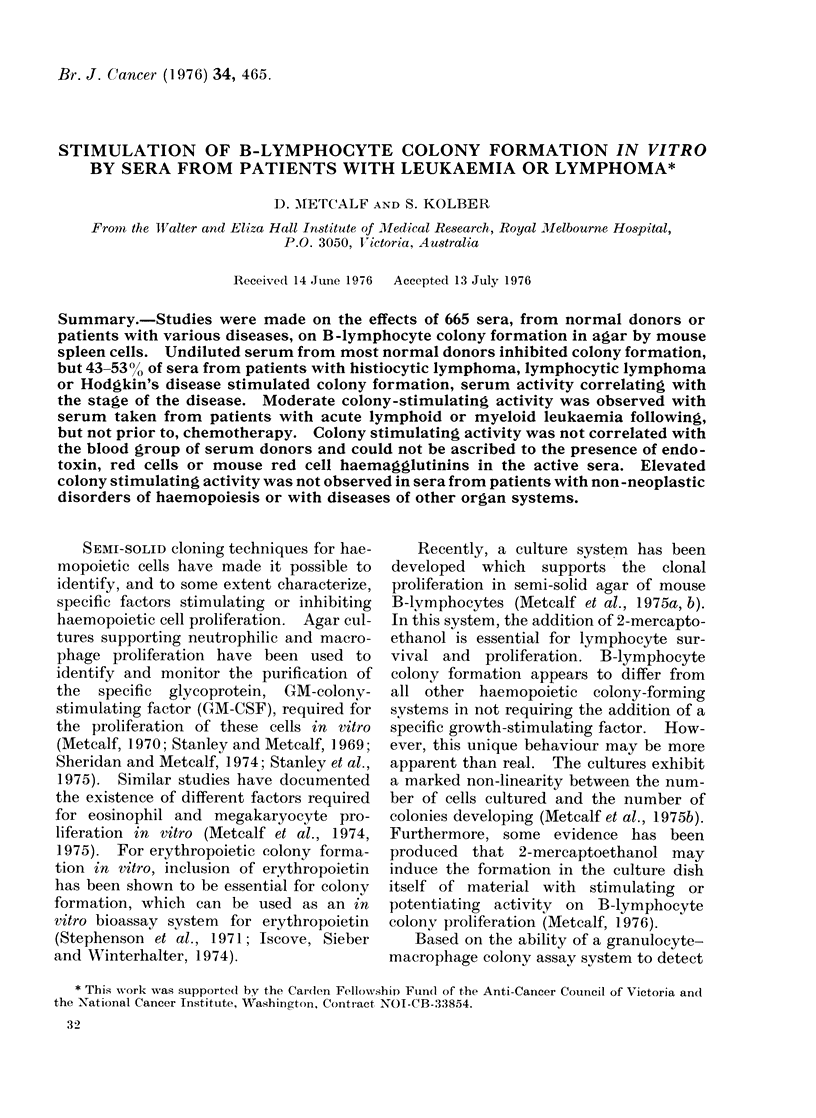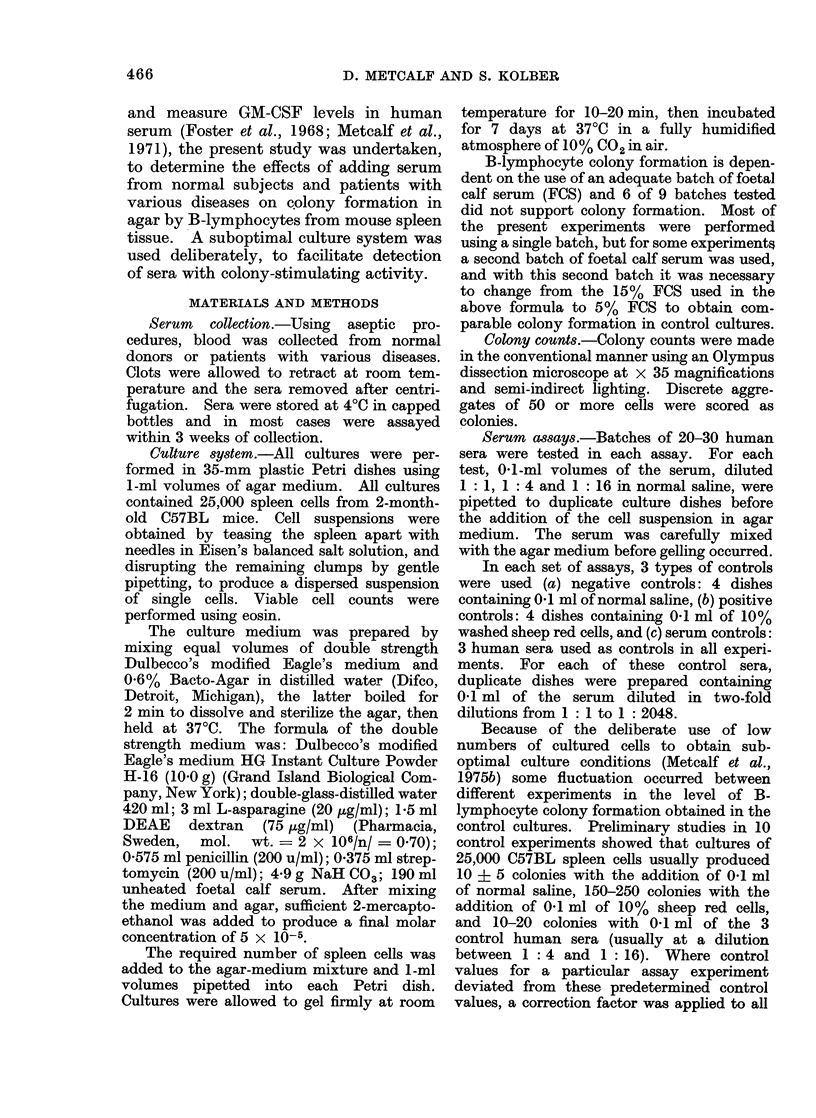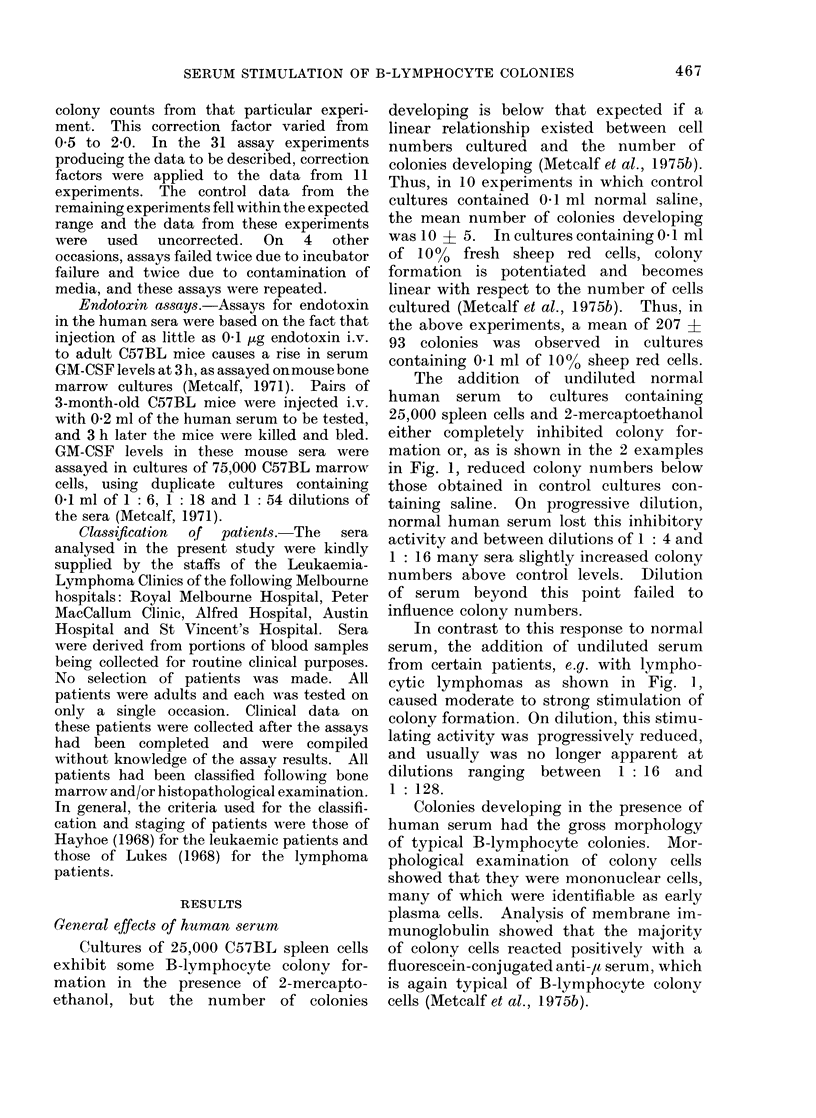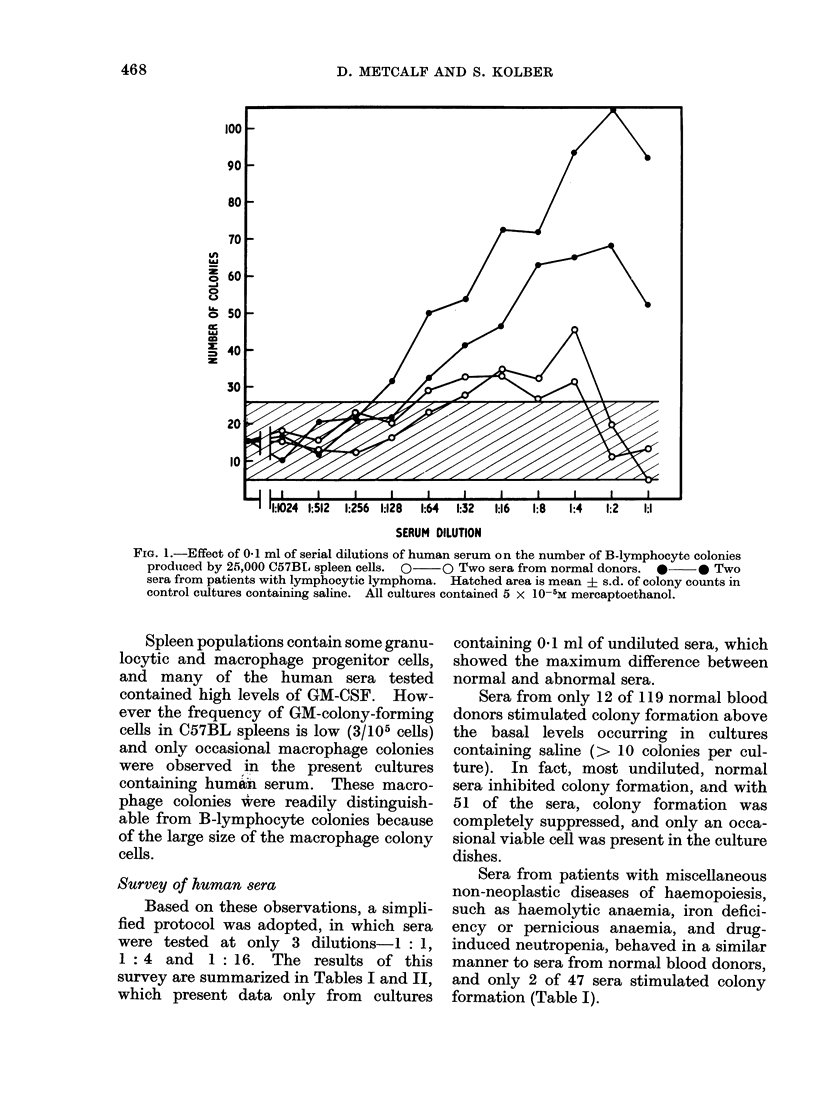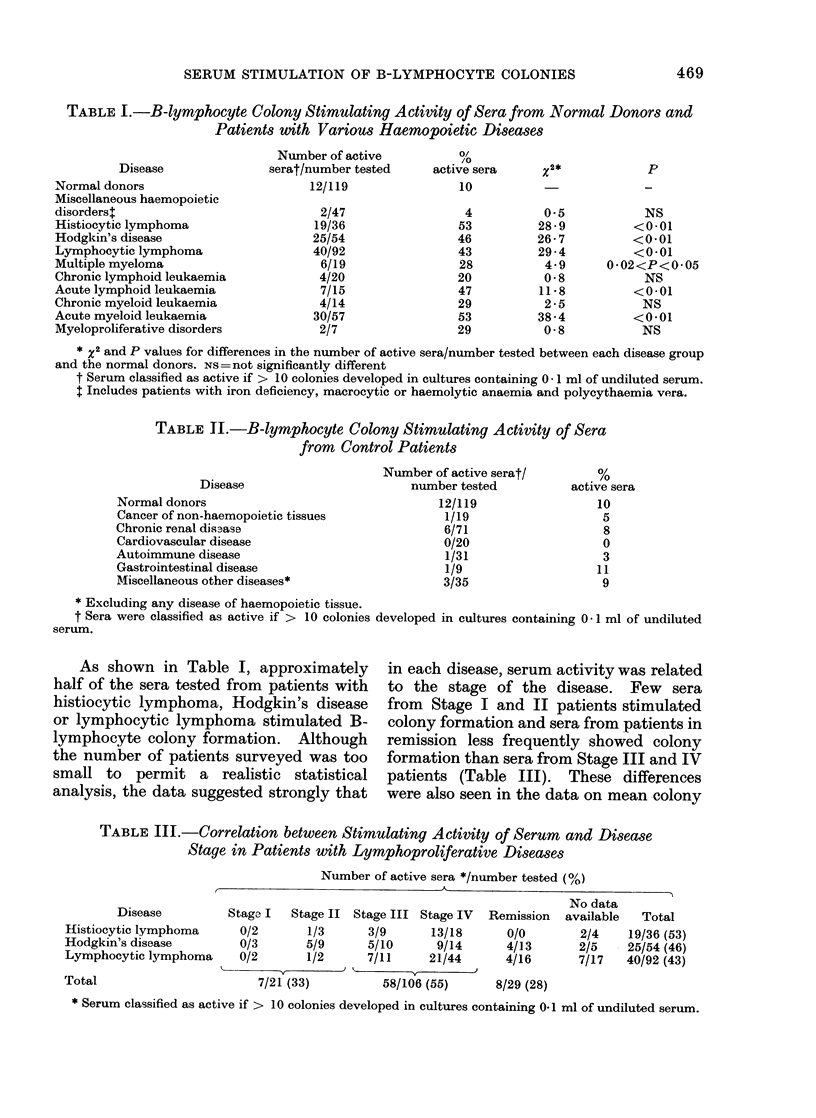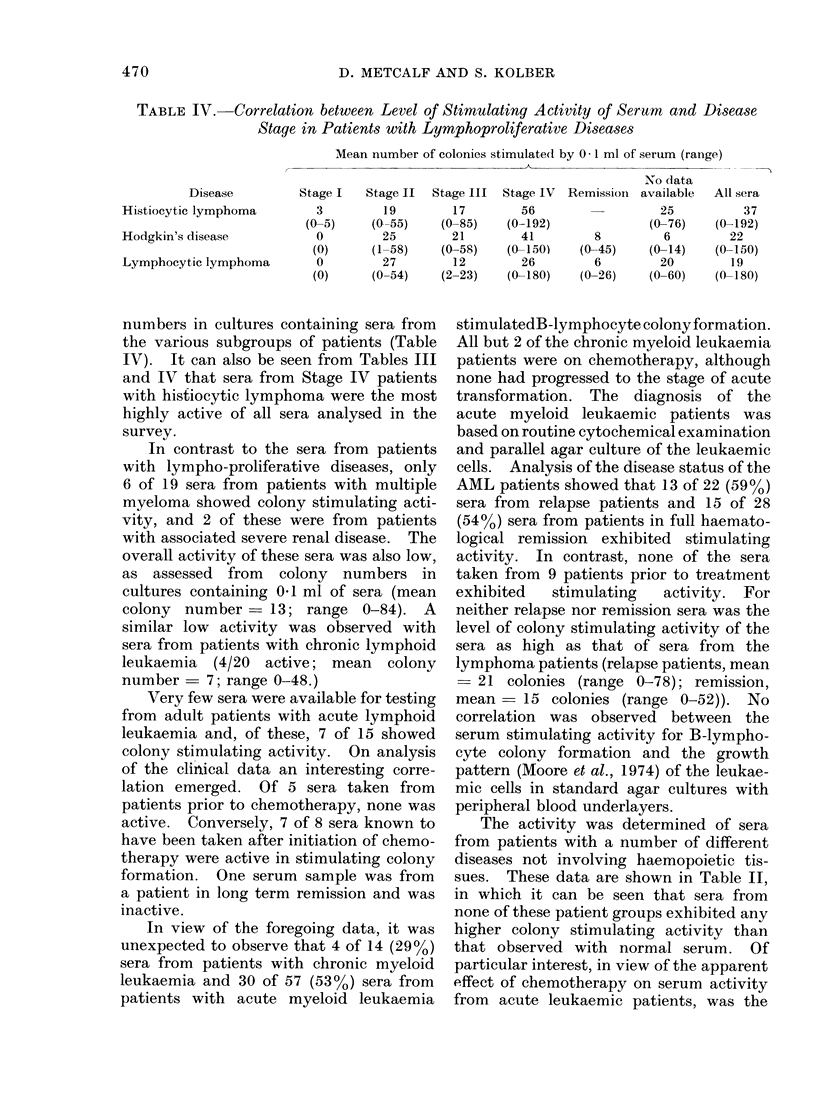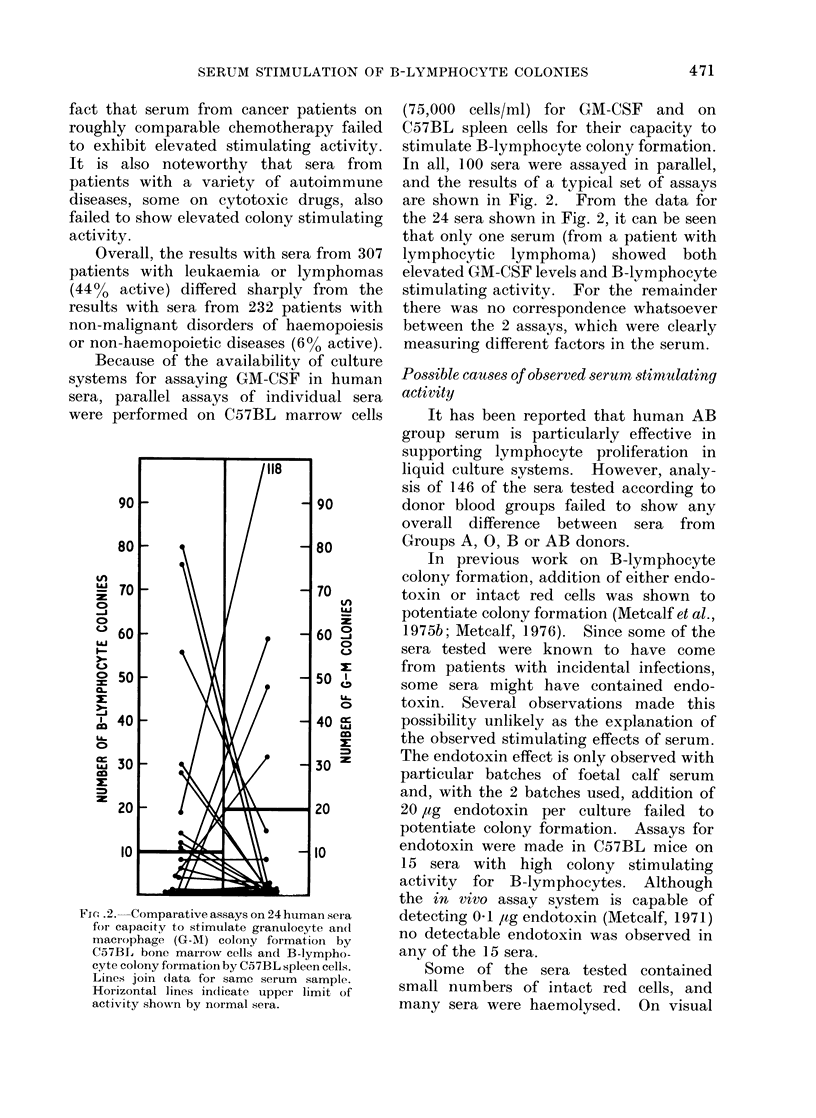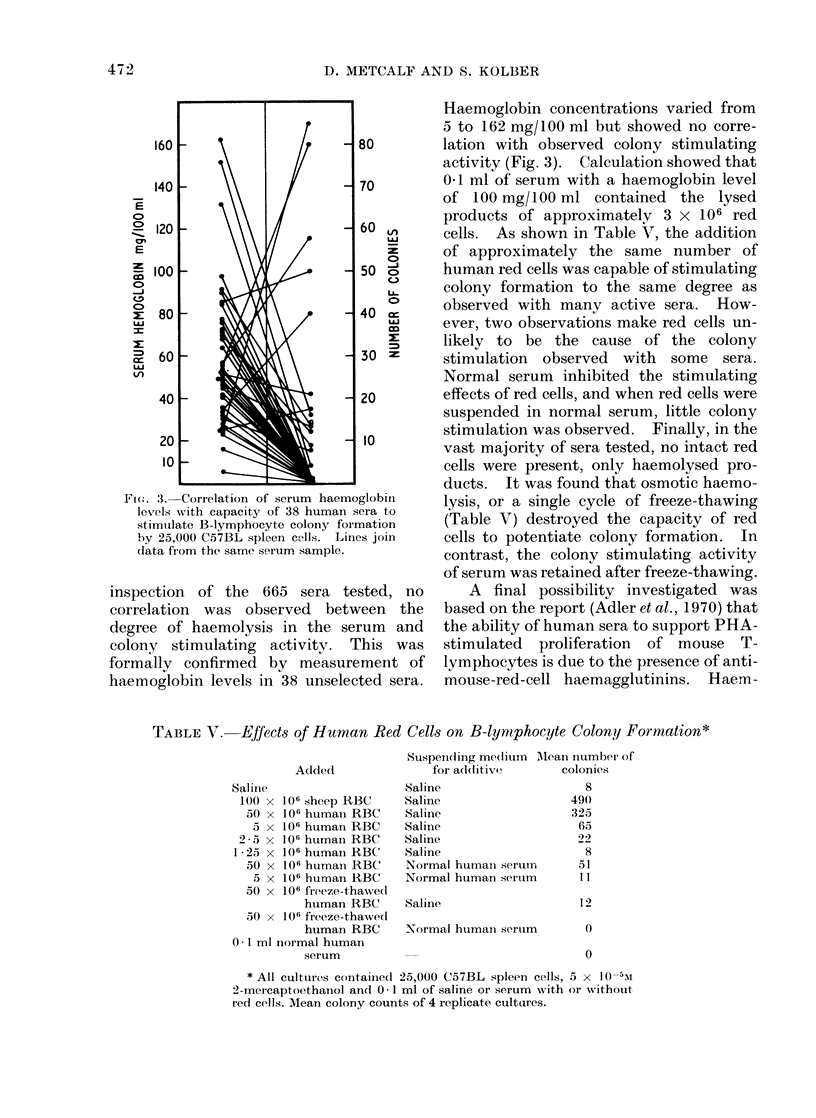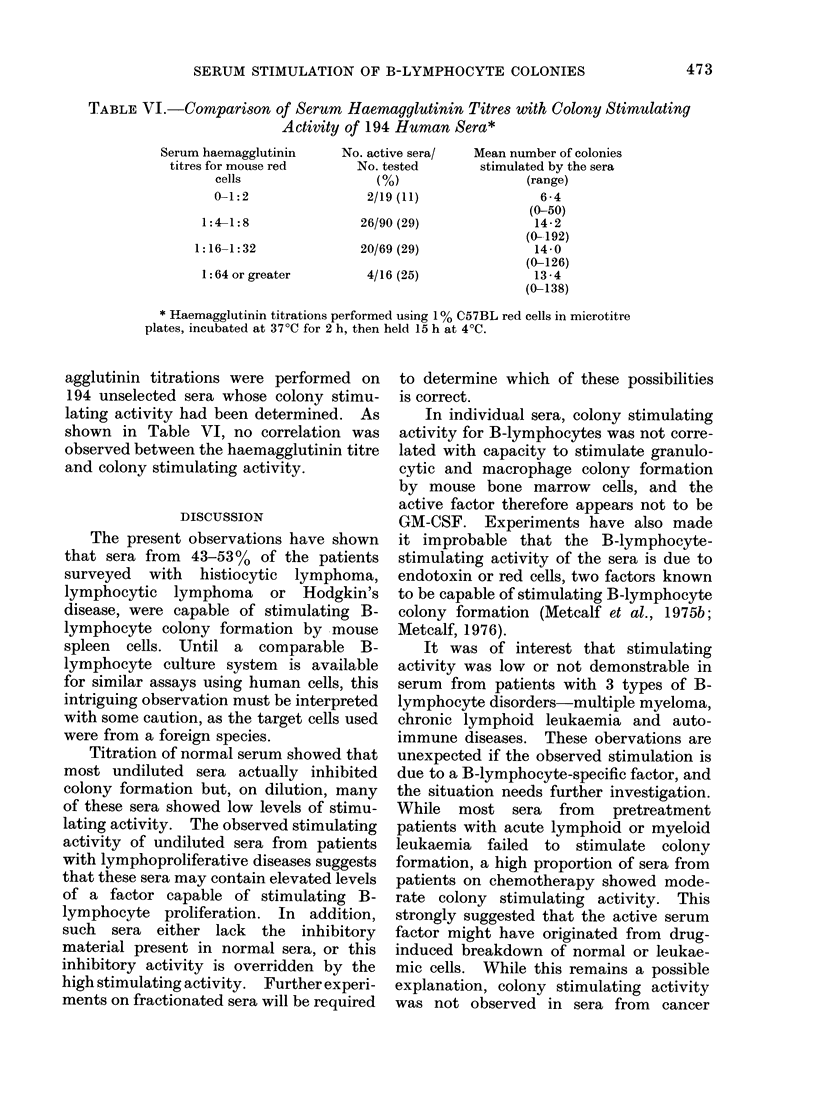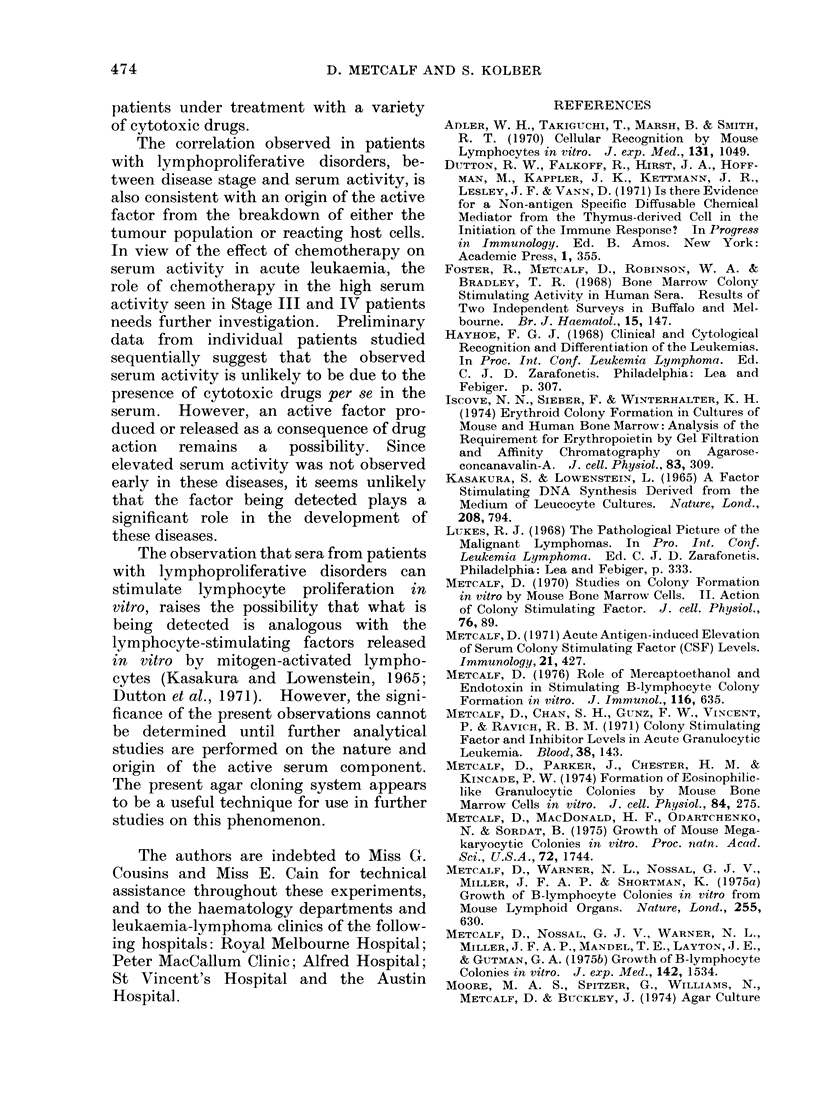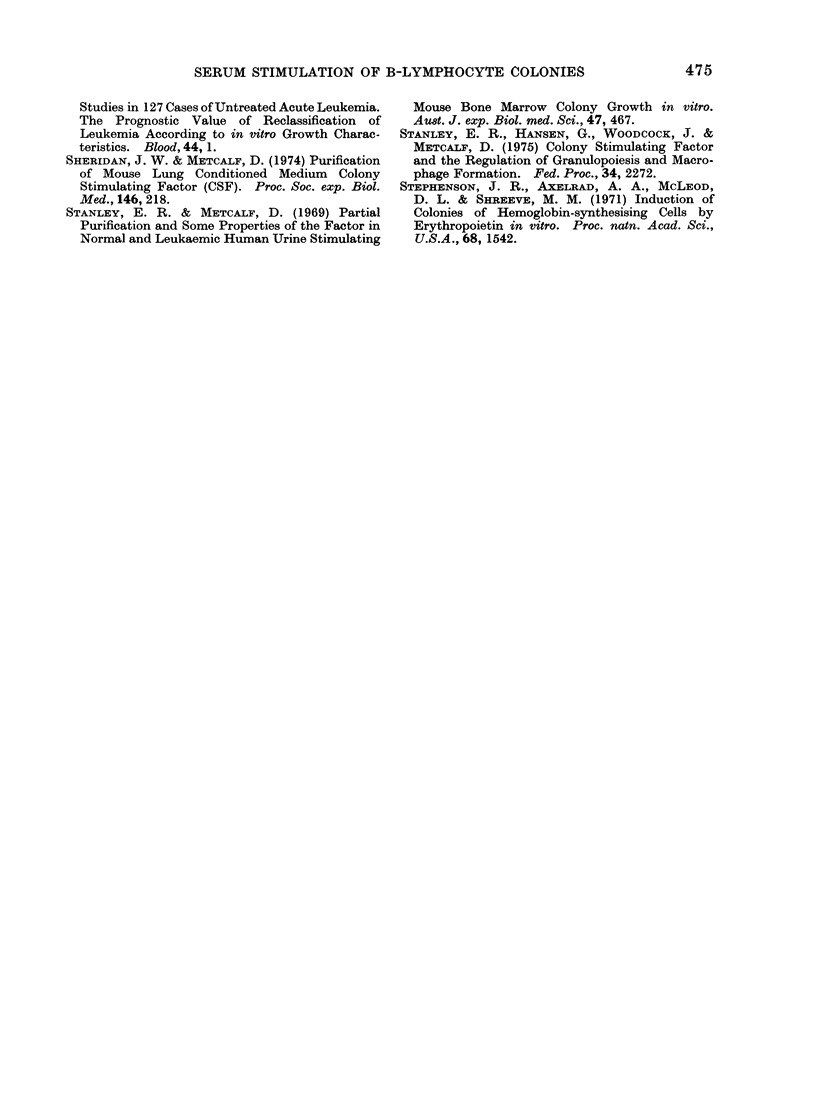# Stimulation of B-lymphocyte colony formation in vitro by sera from patients with leukaemia or lymphoma.

**DOI:** 10.1038/bjc.1976.200

**Published:** 1976-11

**Authors:** D. Metcalf, S. Kolber

## Abstract

Studies were made on the effects of 665 sera, from normal donors or patients with various diseases, on B-lymphocyte colony formation in agar by mouse spleen cells. Undiluted serum from most normal donors inhibited colony formation, but 43-53% of sera from patients with histiocytic lymphoma, lymphocytic lymphoma or Hodgkin's disease stimulated colony formation, serum activity correlating with the stage of the disease. Moderate colony-stimulating activity was observed with serum taken from patients with acute lymphoid or myeloid leukaemia following, but not prior to, chemotherapy. Colony stimulating activity was not correlated with the blood group of serum donors and could not be ascribed to the presence of endotoxin, red cells or mouse red cell haemagglutinins in the active sera. Elevated colony stimulating activity was not observed in sera from patients with non-neoplastic disorders ot haemopoiesis or with diseases of other organ systems.


*Br. J. Cancer* (1976) **34**, 465.

### STIMULATION OF B-LYMPHOCYTE COLONY FORMATION *IN VITRO* BY SERA FROM PATIENTS WITH LEUKAEMIA OR LYMPHOMA*

D. METCALF AND S. KOLBER

*From the Walter and Eliza Hall Institute of Medical Research, Royal Melbourne Hospital, P.O. 3050, Victoria, Australia*

Received 14 June 1976 Accepted 13 July 1976

**Summary.—Studies were made on the effects of 665 sera, from normal donors or patients with various diseases, on B-lymphocyte colony formation in agar by mouse spleen cells. Undiluted serum from most normal donors inhibited colony formation, but 43–53% of sera from patients with histiocytic lymphoma, lymphocytic lymphoma or Hodgkin's disease stimulated colony formation, serum activity correlating with the stage of the disease. Moderate colony-stimulating activity was observed with serum taken from patients with acute lymphoid or myeloid leukaemia following, but not prior to, chemotherapy. Colony stimulating activity was not correlated with the blood group of serum donors and could not be ascribed to the presence of endotoxin, red cells or mouse red cell haemagglutinins in the active sera. Elevated colony stimulating activity was not observed in sera from patients with non-neoplastic disorders of haemopoiesis or with diseases of other organ systems.**

SEMI-SOLID cloning techniques for haemopoietic cells have made it possible to identify, and to some extent characterize, specific factors stimulating or inhibiting haemopoietic cell proliferation. Agar cultures supporting neutrophilic and macrophage proliferation have been used to identify and monitor the purification of the specific glycoprotein, GM-colony-stimulating factor (GM-CSF), required for the proliferation of these cells *in vitro* (Metcalf, 1970; Stanley and Metcalf, 1969; Sheridan and Metcalf, 1974; Stanley *et al.*, 1975). Similar studies have documented the existence of different factors required for eosinophil and megakaryocyte proliferation *in vitro* (Metcalf *et al.*, 1974, 1975). For erythropoietic colony formation *in vitro*, inclusion of erythropoietin has been shown to be essential for colony formation, which can be used as an *in vitro* bioassay system for erythropoietin (Stephenson *et al.*, 1971; Iscove, Sieber and Winterhalter, 1974).

Recently, a culture system has been developed which supports the clonal proliferation in semi-solid agar of mouse B-lymphocytes (Metcalf *et al.*, 1975*a, b*). In this system, the addition of 2-mercaptoethanol is essential for lymphocyte survival and proliferation. B-lymphocyte colony formation appears to differ from all other haemopoietic colony-forming systems in not requiring the addition of a specific growth-stimulating factor. However, this unique behaviour may be more apparent than real. The cultures exhibit a marked non-linearity between the number of cells cultured and the number of colonies developing (Metcalf *et al.*, 1975*b*). Furthermore, some evidence has been produced that 2-mercaptoethanol may induce the formation in the culture dish itself of material with stimulating or potentiating activity on B-lymphocyte colony proliferation (Metcalf, 1976).

Based on the ability of a granulocyte–macrophage colony assay system to detect

* This work was supported by the Carden Fellowship Fund of the Anti-Cancer Council of Victoria and the National Cancer Institute, Washington, Contract NOI-CB-33854.

and measure GM-CSF levels in human serum (Foster *et al.*, 1968; Metcalf *et al.*, 1971), the present study was undertaken, to determine the effects of adding serum from normal subjects and patients with various diseases on colony formation in agar by B-lymphocytes from mouse spleen tissue. A suboptimal culture system was used deliberately, to facilitate detection of sera with colony-stimulating activity.

### MATERIALS AND METHODS

*Serum collection.*—Using aseptic procedures, blood was collected from normal donors or patients with various diseases. Clots were allowed to retract at room temperature and the sera removed after centrifugation. Sera were stored at 4°C in capped bottles and in most cases were assayed within 3 weeks of collection.

*Culture system.*—All cultures were performed in 35-mm plastic Petri dishes using 1-ml volumes of agar medium. All cultures contained 25,000 spleen cells from 2-month-old C57BL mice. Cell suspensions were obtained by teasing the spleen apart with needles in Eisen's balanced salt solution, and disrupting the remaining clumps by gentle pipetting, to produce a dispersed suspension of single cells. Viable cell counts were performed using eosin.

The culture medium was prepared by mixing equal volumes of double strength Dulbecco's modified Eagle's medium and 0·6% Bacto-Agar in distilled water (Difco, Detroit, Michigan), the latter boiled for 2 min to dissolve and sterilize the agar, then held at 37°C. The formula of the double strength medium was: Dulbecco's modified Eagle's medium HG Instant Culture Powder H-16 (10·0 g) (Grand Island Biological Company, New York); double-glass-distilled water 420 ml; 3 ml L-asparagine (20 $\mu$g/ml); 1·5 ml DEAE dextran (75 $\mu$g/ml) (Pharmacia, Sweden, mol. wt. $= 2 \times 10^6/n/ = 0{\cdot}70$); 0·575 ml penicillin (200 u/ml); 0·375 ml streptomycin (200 u/ml); 4·9 g $NaHCO_3$; 190 ml unheated foetal calf serum. After mixing the medium and agar, sufficient 2-mercaptoethanol was added to produce a final molar concentration of $5 \times 10^{-5}$.

The required number of spleen cells was added to the agar-medium mixture and 1-ml volumes pipetted into each Petri dish. Cultures were allowed to gel firmly at room temperature for 10–20 min, then incubated for 7 days at 37°C in a fully humidified atmosphere of 10% $CO_2$ in air.

B-lymphocyte colony formation is dependent on the use of an adequate batch of foetal calf serum (FCS) and 6 of 9 batches tested did not support colony formation. Most of the present experiments were performed using a single batch, but for some experiments a second batch of foetal calf serum was used, and with this second batch it was necessary to change from the 15% FCS used in the above formula to 5% FCS to obtain comparable colony formation in control cultures.

*Colony counts.*—Colony counts were made in the conventional manner using an Olympus dissection microscope at × 35 magnifications and semi-indirect lighting. Discrete aggregates of 50 or more cells were scored as colonies.

*Serum assays.*—Batches of 20–30 human sera were tested in each assay. For each test, 0·1-ml volumes of the serum, diluted 1 : 1, 1 : 4 and 1 : 16 in normal saline, were pipetted to duplicate culture dishes before the addition of the cell suspension in agar medium. The serum was carefully mixed with the agar medium before gelling occurred.

In each set of assays, 3 types of controls were used (*a*) negative controls: 4 dishes containing 0·1 ml of normal saline, (*b*) positive controls: 4 dishes containing 0·1 ml of 10% washed sheep red cells, and (*c*) serum controls: 3 human sera used as controls in all experiments. For each of these control sera, duplicate dishes were prepared containing 0·1 ml of the serum diluted in two-fold dilutions from 1 : 1 to 1 : 2048.

Because of the deliberate use of low numbers of cultured cells to obtain suboptimal culture conditions (Metcalf *et al.*, 1975*b*) some fluctuation occurred between different experiments in the level of B-lymphocyte colony formation obtained in the control cultures. Preliminary studies in 10 control experiments showed that cultures of 25,000 C57BL spleen cells usually produced 10 ± 5 colonies with the addition of 0·1 ml of normal saline, 150–250 colonies with the addition of 0·1 ml of 10% sheep red cells, and 10–20 colonies with 0·1 ml of the 3 control human sera (usually at a dilution between 1 : 4 and 1 : 16). Where control values for a particular assay experiment deviated from these predetermined control values, a correction factor was applied to all colony counts from that particular experiment. This correction factor varied from 0·5 to 2·0. In the 31 assay experiments producing the data to be described, correction factors were applied to the data from 11 experiments. The control data from the remaining experiments fell within the expected range and the data from these experiments were used uncorrected. On 4 other occasions, assays failed twice due to incubator failure and twice due to contamination of media, and these assays were repeated.

*Endotoxin assays.*—Assays for endotoxin in the human sera were based on the fact that injection of as little as 0·1 $\mu$g endotoxin i.v. to adult C57BL mice causes a rise in serum GM-CSF levels at 3 h, as assayed on mouse bone marrow cultures (Metcalf, 1971). Pairs of 3-month-old C57BL mice were injected i.v. with 0·2 ml of the human serum to be tested, and 3 h later the mice were killed and bled. GM-CSF levels in these mouse sera were assayed in cultures of 75,000 C57BL marrow cells, using duplicate cultures containing 0·1 ml of 1 : 6, 1 : 18 and 1 : 54 dilutions of the sera (Metcalf, 1971).

*Classification of patients.*—The sera analysed in the present study were kindly supplied by the staffs of the Leukaemia-Lymphoma Clinics of the following Melbourne hospitals: Royal Melbourne Hospital, Peter MacCallum Clinic, Alfred Hospital, Austin Hospital and St Vincent's Hospital. Sera were derived from portions of blood samples being collected for routine clinical purposes. No selection of patients was made. All patients were adults and each was tested on only a single occasion. Clinical data on these patients were collected after the assays had been completed and were compiled without knowledge of the assay results. All patients had been classified following bone marrow and/or histopathological examination. In general, the criteria used for the classification and staging of patients were those of Hayhoe (1968) for the leukaemic patients and those of Lukes (1968) for the lymphoma patients.

### RESULTS

### *General effects of human serum*

Cultures of 25,000 C57BL spleen cells exhibit some B-lymphocyte colony formation in the presence of 2-mercaptoethanol, but the number of colonies developing is below that expected if a linear relationship existed between cell numbers cultured and the number of colonies developing (Metcalf *et al.*, 1975*b*). Thus, in 10 experiments in which control cultures contained 0·1 ml normal saline, the mean number of colonies developing was 10 $\pm$ 5. In cultures containing 0·1 ml of 10% fresh sheep red cells, colony formation is potentiated and becomes linear with respect to the number of cells cultured (Metcalf *et al.*, 1975*b*). Thus, in the above experiments, a mean of 207 $\pm$ 93 colonies was observed in cultures containing 0·1 ml of 10% sheep red cells.

The addition of undiluted normal human serum to cultures containing 25,000 spleen cells and 2-mercaptoethanol either completely inhibited colony formation or, as is shown in the 2 examples in Fig. 1, reduced colony numbers below those obtained in control cultures containing saline. On progressive dilution, normal human serum lost this inhibitory activity and between dilutions of 1 : 4 and 1 : 16 many sera slightly increased colony numbers above control levels. Dilution of serum beyond this point failed to influence colony numbers.

In contrast to this response to normal serum, the addition of undiluted serum from certain patients, *e.g.* with lymphocytic lymphomas as shown in Fig. 1, caused moderate to strong stimulation of colony formation. On dilution, this stimulating activity was progressively reduced, and usually was no longer apparent at dilutions ranging between 1 : 16 and 1 : 128.

Colonies developing in the presence of human serum had the gross morphology of typical B-lymphocyte colonies. Morphological examination of colony cells showed that they were mononuclear cells, many of which were identifiable as early plasma cells. Analysis of membrane immunoglobulin showed that the majority of colony cells reacted positively with a fluorescein-conjugated anti-$\mu$ serum, which is again typical of B-lymphocyte colony cells (Metcalf *et al.*, 1975*b*).

FIG. 1.—Effect of 0·1 ml of serial dilutions of human serum on the number of B-lymphocyte colonies produced by 25,000 C57BL spleen cells. ○——○ Two sera from normal donors. ●——● Two sera from patients with lymphocytic lymphoma. Hatched area is mean ± s.d. of colony counts in control cultures containing saline. All cultures contained $5 \times 10^{-5}$M mercaptoethanol.

Spleen populations contain some granulocytic and macrophage progenitor cells, and many of the human sera tested contained high levels of GM-CSF. However the frequency of GM-colony-forming cells in C57BL spleens is low ($3/10^5$ cells) and only occasional macrophage colonies were observed in the present cultures containing human serum. These macrophage colonies were readily distinguishable from B-lymphocyte colonies because of the large size of the macrophage colony cells.

*Survey of human sera*

Based on these observations, a simplified protocol was adopted, in which sera were tested at only 3 dilutions—1 : 1, 1 : 4 and 1 : 16. The results of this survey are summarized in Tables I and II, which present data only from cultures containing 0·1 ml of undiluted sera, which showed the maximum difference between normal and abnormal sera.

Sera from only 12 of 119 normal blood donors stimulated colony formation above the basal levels occurring in cultures containing saline (> 10 colonies per culture). In fact, most undiluted, normal sera inhibited colony formation, and with 51 of the sera, colony formation was completely suppressed, and only an occasional viable cell was present in the culture dishes.

Sera from patients with miscellaneous non-neoplastic diseases of haemopoiesis, such as haemolytic anaemia, iron deficiency or pernicious anaemia, and drug-induced neutropenia, behaved in a similar manner to sera from normal blood donors, and only 2 of 47 sera stimulated colony formation (Table I).

TABLE I.—*B-lymphocyte Colony Stimulating Activity of Sera from Normal Donors and Patients with Various Haemopoietic Diseases*

| Disease | Number of active sera†/number tested | % active sera | $\chi^2$* | $P$ |
|---|---|---|---|---|
| Normal donors | 12/119 | 10 | — | - |
| Miscellaneous haemopoietic disorders‡ | 2/47 | 4 | 0·5 | NS |
| Histiocytic lymphoma | 19/36 | 53 | 28·9 | <0·01 |
| Hodgkin's disease | 25/54 | 46 | 26·7 | <0·01 |
| Lymphocytic lymphoma | 40/92 | 43 | 29·4 | <0·01 |
| Multiple myeloma | 6/19 | 28 | 4·9 | $0·02<P<0·05$ |
| Chronic lymphoid leukaemia | 4/20 | 20 | 0·8 | NS |
| Acute lymphoid leukaemia | 7/15 | 47 | 11·8 | <0·01 |
| Chronic myeloid leukaemia | 4/14 | 29 | 2·5 | NS |
| Acute myeloid leukaemia | 30/57 | 53 | 38·4 | <0·01 |
| Myeloproliferative disorders | 2/7 | 29 | 0·8 | NS |

* $\chi^2$ and $P$ values for differences in the number of active sera/number tested between each disease group and the normal donors. NS = not significantly different

† Serum classified as active if > 10 colonies developed in cultures containing 0·1 ml of undiluted serum.

‡ Includes patients with iron deficiency, macrocytic or haemolytic anaemia and polycythaemia vera.

TABLE II.—*B-lymphocyte Colony Stimulating Activity of Sera from Control Patients*

| Disease | Number of active sera†/number tested | % active sera |
|---|---|---|
| Normal donors | 12/119 | 10 |
| Cancer of non-haemopoietic tissues | 1/19 | 5 |
| Chronic renal disease | 6/71 | 8 |
| Cardiovascular disease | 0/20 | 0 |
| Autoimmune disease | 1/31 | 3 |
| Gastrointestinal disease | 1/9 | 11 |
| Miscellaneous other diseases* | 3/35 | 9 |

* Excluding any disease of haemopoietic tissue.

† Sera were classified as active if > 10 colonies developed in cultures containing 0·1 ml of undiluted serum.

As shown in Table I, approximately half of the sera tested from patients with histiocytic lymphoma, Hodgkin's disease or lymphocytic lymphoma stimulated B-lymphocyte colony formation. Although the number of patients surveyed was too small to permit a realistic statistical analysis, the data suggested strongly that in each disease, serum activity was related to the stage of the disease. Few sera from Stage I and II patients stimulated colony formation and sera from patients in remission less frequently showed colony formation than sera from Stage III and IV patients (Table III). These differences were also seen in the data on mean colony

TABLE III.—*Correlation between Stimulating Activity of Serum and Disease Stage in Patients with Lymphoproliferative Diseases*

| Disease | Number of active sera */number tested (%) | | | | | | |
|---|---|---|---|---|---|---|---|
| | Stage I | Stage II | Stage III | Stage IV | Remission | No data available | Total |
| Histiocytic lymphoma | 0/2 | 1/3 | 3/9 | 13/18 | 0/0 | 2/4 | 19/36 (53) |
| Hodgkin's disease | 0/3 | 5/9 | 5/10 | 9/14 | 4/13 | 2/5 | 25/54 (46) |
| Lymphocytic lymphoma | 0/2 | 1/2 | 7/11 | 21/44 | 4/16 | 7/17 | 40/92 (43) |
| Total | 7/21 (33) | | 58/106 (55) | | 8/29 (28) | | |

* Serum classified as active if > 10 colonies developed in cultures containing 0·1 ml of undiluted serum.

TABLE IV.—*Correlation between Level of Stimulating Activity of Serum and Disease Stage in Patients with Lymphoproliferative Diseases*

| Disease | Mean number of colonies stimulated by 0·1 ml of serum (range) | | | | | | |
|---|---|---|---|---|---|---|---|
| | Stage I | Stage II | Stage III | Stage IV | Remission | No data available | All sera |
| Histiocytic lymphoma | 3 (0–5) | 19 (0–55) | 17 (0–85) | 56 (0–192) | — | 25 (0–76) | 37 (0–192) |
| Hodgkin's disease | 0 (0) | 25 (1–58) | 21 (0–58) | 41 (0–150) | 8 (0–45) | 6 (0–14) | 22 (0–150) |
| Lymphocytic lymphoma | 0 (0) | 27 (0–54) | 12 (2–23) | 26 (0–180) | 6 (0–26) | 20 (0–60) | 19 (0–180) |

numbers in cultures containing sera from the various subgroups of patients (Table IV). It can also be seen from Tables III and IV that sera from Stage IV patients with histiocytic lymphoma were the most highly active of all sera analysed in the survey.

In contrast to the sera from patients with lympho-proliferative diseases, only 6 of 19 sera from patients with multiple myeloma showed colony stimulating activity, and 2 of these were from patients with associated severe renal disease. The overall activity of these sera was also low, as assessed from colony numbers in cultures containing 0·1 ml of sera (mean colony number = 13; range 0–84). A similar low activity was observed with sera from patients with chronic lymphoid leukaemia (4/20 active; mean colony number = 7; range 0–48.)

Very few sera were available for testing from adult patients with acute lymphoid leukaemia and, of these, 7 of 15 showed colony stimulating activity. On analysis of the clinical data an interesting correlation emerged. Of 5 sera taken from patients prior to chemotherapy, none was active. Conversely, 7 of 8 sera known to have been taken after initiation of chemotherapy were active in stimulating colony formation. One serum sample was from a patient in long term remission and was inactive.

In view of the foregoing data, it was unexpected to observe that 4 of 14 (29%) sera from patients with chronic myeloid leukaemia and 30 of 57 (53%) sera from patients with acute myeloid leukaemia stimulated B-lymphocyte colony formation. All but 2 of the chronic myeloid leukaemia patients were on chemotherapy, although none had progressed to the stage of acute transformation. The diagnosis of the acute myeloid leukaemic patients was based on routine cytochemical examination and parallel agar culture of the leukaemic cells. Analysis of the disease status of the AML patients showed that 13 of 22 (59%) sera from relapse patients and 15 of 28 (54%) sera from patients in full haematological remission exhibited stimulating activity. In contrast, none of the sera taken from 9 patients prior to treatment exhibited stimulating activity. For neither relapse nor remission sera was the level of colony stimulating activity of the sera as high as that of sera from the lymphoma patients (relapse patients, mean = 21 colonies (range 0–78); remission, mean = 15 colonies (range 0–52)). No correlation was observed between the serum stimulating activity for B-lymphocyte colony formation and the growth pattern (Moore *et al.*, 1974) of the leukaemic cells in standard agar cultures with peripheral blood underlayers.

The activity was determined of sera from patients with a number of different diseases not involving haemopoietic tissues. These data are shown in Table II, in which it can be seen that sera from none of these patient groups exhibited any higher colony stimulating activity than that observed with normal serum. Of particular interest, in view of the apparent effect of chemotherapy on serum activity from acute leukaemic patients, was the fact that serum from cancer patients on roughly comparable chemotherapy failed to exhibit elevated stimulating activity. It is also noteworthy that sera from patients with a variety of autoimmune diseases, some on cytotoxic drugs, also failed to show elevated colony stimulating activity.

Overall, the results with sera from 307 patients with leukaemia or lymphomas (44% active) differed sharply from the results with sera from 232 patients with non-malignant disorders of haemopoiesis or non-haemopoietic diseases (6% active).

Because of the availability of culture systems for assaying GM-CSF in human sera, parallel assays of individual sera were performed on C57BL marrow cells (75,000 cells/ml) for GM-CSF and on C57BL spleen cells for their capacity to stimulate B-lymphocyte colony formation. In all, 100 sera were assayed in parallel, and the results of a typical set of assays are shown in Fig. 2. From the data for the 24 sera shown in Fig. 2, it can be seen that only one serum (from a patient with lymphocytic lymphoma) showed both elevated GM-CSF levels and B-lymphocyte stimulating activity. For the remainder there was no correspondence whatsoever between the 2 assays, which were clearly measuring different factors in the serum.

FIG. 2.—Comparative assays on 24 human sera for capacity to stimulate granulocyte and macrophage (G-M) colony formation by C57BL bone marrow cells and B-lymphocyte colony formation by C57BL spleen cells. Lines join data for same serum sample. Horizontal lines indicate upper limit of activity shown by normal sera.

*Possible causes of observed serum stimulating activity*

It has been reported that human AB group serum is particularly effective in supporting lymphocyte proliferation in liquid culture systems. However, analysis of 146 of the sera tested according to donor blood groups failed to show any overall difference between sera from Groups A, O, B or AB donors.

In previous work on B-lymphocyte colony formation, addition of either endotoxin or intact red cells was shown to potentiate colony formation (Metcalf *et al.*, 1975*b*; Metcalf, 1976). Since some of the sera tested were known to have come from patients with incidental infections, some sera might have contained endotoxin. Several observations made this possibility unlikely as the explanation of the observed stimulating effects of serum. The endotoxin effect is only observed with particular batches of foetal calf serum and, with the 2 batches used, addition of 20 $\mu$g endotoxin per culture failed to potentiate colony formation. Assays for endotoxin were made in C57BL mice on 15 sera with high colony stimulating activity for B-lymphocytes. Although the *in vivo* assay system is capable of detecting 0·1 $\mu$g endotoxin (Metcalf, 1971) no detectable endotoxin was observed in any of the 15 sera.

Some of the sera tested contained small numbers of intact red cells, and many sera were haemolysed. On visual

FIG. 3.—Correlation of serum haemoglobin levels with capacity of 38 human sera to stimulate B-lymphocyte colony formation by 25,000 C57BL spleen cells. Lines join data from the same serum sample.

inspection of the 665 sera tested, no correlation was observed between the degree of haemolysis in the serum and colony stimulating activity. This was formally confirmed by measurement of haemoglobin levels in 38 unselected sera. Haemoglobin concentrations varied from 5 to 162 mg/100 ml but showed no correlation with observed colony stimulating activity (Fig. 3). Calculation showed that 0·1 ml of serum with a haemoglobin level of 100 mg/100 ml contained the lysed products of approximately $3 \times 10^6$ red cells. As shown in Table V, the addition of approximately the same number of human red cells was capable of stimulating colony formation to the same degree as observed with many active sera. However, two observations make red cells unlikely to be the cause of the colony stimulation observed with some sera. Normal serum inhibited the stimulating effects of red cells, and when red cells were suspended in normal serum, little colony stimulation was observed. Finally, in the vast majority of sera tested, no intact red cells were present, only haemolysed products. It was found that osmotic haemolysis, or a single cycle of freeze-thawing (Table V) destroyed the capacity of red cells to potentiate colony formation. In contrast, the colony stimulating activity of serum was retained after freeze-thawing.

A final possibility investigated was based on the report (Adler *et al.*, 1970) that the ability of human sera to support PHA-stimulated proliferation of mouse T-lymphocytes is due to the presence of anti-mouse-red-cell haemagglutinins. Haem-

TABLE V.—*Effects of Human Red Cells on B-lymphocyte Colony Formation**

| Added | Suspending medium for additive | Mean number of colonies |
|---|---|---|
| Saline | Saline | 8 |
| $100 \times 10^6$ sheep RBC | Saline | 490 |
| $50 \times 10^6$ human RBC | Saline | 325 |
| $5 \times 10^6$ human RBC | Saline | 65 |
| $2 \cdot 5 \times 10^6$ human RBC | Saline | 22 |
| $1 \cdot 25 \times 10^6$ human RBC | Saline | 8 |
| $50 \times 10^6$ human RBC | Normal human serum | 51 |
| $5 \times 10^6$ human RBC | Normal human serum | 11 |
| $50 \times 10^6$ freeze-thawed human RBC | Saline | 12 |
| $50 \times 10^6$ freeze-thawed human RBC | Normal human serum | 0 |
| 0·1 ml normal human serum | — | 0 |

* All cultures contained 25,000 C57BL spleen cells, $5 \times 10^{-5}$M 2-mercaptoethanol and 0·1 ml of saline or serum with or without red cells. Mean colony counts of 4 replicate cultures.

TABLE VI.—*Comparison of Serum Haemagglutinin Titres with Colony Stimulating Activity of* 194 *Human Sera**

| Serum haemagglutinin titres for mouse red cells | No. active sera/ No. tested (%) | Mean number of colonies stimulated by the sera (range) |
|---|---|---|
| 0–1:2 | 2/19 (11) | 6·4 (0–50) |
| 1:4–1:8 | 26/90 (29) | 14·2 (0–192) |
| 1:16–1:32 | 20/69 (29) | 14·0 (0–126) |
| 1:64 or greater | 4/16 (25) | 13·4 (0–138) |

* Haemagglutinin titrations performed using 1% C57BL red cells in microtitre plates, incubated at 37°C for 2 h, then held 15 h at 4°C.

agglutinin titrations were performed on 194 unselected sera whose colony stimulating activity had been determined. As shown in Table VI, no correlation was observed between the haemagglutinin titre and colony stimulating activity.

### DISCUSSION

The present observations have shown that sera from 43–53% of the patients surveyed with histiocytic lymphoma, lymphocytic lymphoma or Hodgkin's disease, were capable of stimulating B-lymphocyte colony formation by mouse spleen cells. Until a comparable B-lymphocyte culture system is available for similar assays using human cells, this intriguing observation must be interpreted with some caution, as the target cells used were from a foreign species.

Titration of normal serum showed that most undiluted sera actually inhibited colony formation but, on dilution, many of these sera showed low levels of stimulating activity. The observed stimulating activity of undiluted sera from patients with lymphoproliferative diseases suggests that these sera may contain elevated levels of a factor capable of stimulating B-lymphocyte proliferation. In addition, such sera either lack the inhibitory material present in normal sera, or this inhibitory activity is overridden by the high stimulating activity. Further experiments on fractionated sera will be required to determine which of these possibilities is correct.

In individual sera, colony stimulating activity for B-lymphocytes was not correlated with capacity to stimulate granulocytic and macrophage colony formation by mouse bone marrow cells, and the active factor therefore appears not to be GM-CSF. Experiments have also made it improbable that the B-lymphocyte-stimulating activity of the sera is due to endotoxin or red cells, two factors known to be capable of stimulating B-lymphocyte colony formation (Metcalf *et al.*, 1975*b*; Metcalf, 1976).

It was of interest that stimulating activity was low or not demonstrable in serum from patients with 3 types of B-lymphocyte disorders—multiple myeloma, chronic lymphoid leukaemia and autoimmune diseases. These obervations are unexpected if the observed stimulation is due to a B-lymphocyte-specific factor, and the situation needs further investigation. While most sera from pretreatment patients with acute lymphoid or myeloid leukaemia failed to stimulate colony formation, a high proportion of sera from patients on chemotherapy showed moderate colony stimulating activity. This strongly suggested that the active serum factor might have originated from drug-induced breakdown of normal or leukaemic cells. While this remains a possible explanation, colony stimulating activity was not observed in sera from cancer patients under treatment with a variety of cytotoxic drugs.

The correlation observed in patients with lymphoproliferative disorders, between disease stage and serum activity, is also consistent with an origin of the active factor from the breakdown of either the tumour population or reacting host cells. In view of the effect of chemotherapy on serum activity in acute leukaemia, the role of chemotherapy in the high serum activity seen in Stage III and IV patients needs further investigation. Preliminary data from individual patients studied sequentially suggest that the observed serum activity is unlikely to be due to the presence of cytotoxic drugs *per se* in the serum. However, an active factor produced or released as a consequence of drug action remains a possibility. Since elevated serum activity was not observed early in these diseases, it seems unlikely that the factor being detected plays a significant role in the development of these diseases.

The observation that sera from patients with lymphoproliferative disorders can stimulate lymphocyte proliferation *in vitro*, raises the possibility that what is being detected is analogous with the lymphocyte-stimulating factors released *in vitro* by mitogen-activated lymphocytes (Kasakura and Lowenstein, 1965; Dutton *et al.*, 1971). However, the significance of the present observations cannot be determined until further analytical studies are performed on the nature and origin of the active serum component. The present agar cloning system appears to be a useful technique for use in further studies on this phenomenon.

The authors are indebted to Miss G. Cousins and Miss E. Cain for technical assistance throughout these experiments, and to the haematology departments and leukaemia-lymphoma clinics of the following hospitals: Royal Melbourne Hospital; Peter MacCallum Clinic; Alfred Hospital; St Vincent's Hospital and the Austin Hospital.

REFERENCES

ADLER, W. H., TAKIGUCHI, T., MARSH, B. & SMITH, R. T. (1970) Cellular Recognition by Mouse Lymphocytes *in vitro*. *J. exp. Med.*, **131**, 1049.

DUTTON, R. W., FALKOFF, R., HIRST, J. A., HOFFMAN, M., KAPPLER, J. K., KETTMANN, J. R., LESLEY, J. F. & VANN, D. (1971) Is there Evidence for a Non-antigen Specific Diffusable Chemical Mediator from the Thymus-derived Cell in the Initiation of the Immune Response? In *Progress in Immunology*. Ed. B. Amos. New York: Academic Press, **1**, 355.

FOSTER, R., METCALF, D., ROBINSON, W. A. & BRADLEY, T. R. (1968) Bone Marrow Colony Stimulating Activity in Human Sera. Results of Two Independent Surveys in Buffalo and Melbourne. *Br. J. Haematol.*, **15**, 147.

HAYHOE, F. G. J. (1968) Clinical and Cytological Recognition and Differentiation of the Leukemias. In *Proc. Int. Conf. Leukemia Lymphoma*. Ed. C. J. D. Zarafonetis. Philadelphia: Lea and Febiger. p. 307.

ISCOVE, N. N., SIEBER, F. & WINTERHALTER, K. H. (1974) Erythroid Colony Formation in Cultures of Mouse and Human Bone Marrow: Analysis of the Requirement for Erythropoietin by Gel Filtration and Affinity Chromatography on Agarose-concanavalin-A. *J. cell. Physiol.*, **83**, 309.

KASAKURA, S. & LOWENSTEIN, L. (1965) A Factor Stimulating DNA Synthesis Derived from the Medium of Leucocyte Cultures. *Nature, Lond.*, **208**, 794.

LUKES, R. J. (1968) The Pathological Picture of the Malignant Lymphomas. In *Pro. Int. Conf. Leukemia Lymphoma*. Ed. C. J. D. Zarafonetis. Philadelphia: Lea and Febiger, p. 333.

METCALF, D. (1970) Studies on Colony Formation *in vitro* by Mouse Bone Marrow Cells. II. Action of Colony Stimulating Factor. *J. cell. Physiol.*, **76**, 89.

METCALF, D. (1971) Acute Antigen-induced Elevation of Serum Colony Stimulating Factor (CSF) Levels. *Immunology*, **21**, 427.

METCALF, D. (1976) Role of Mercaptoethanol and Endotoxin in Stimulating B-lymphocyte Colony Formation *in vitro*. *J. Immunol.*, **116**, 635.

METCALF, D., CHAN, S. H., GUNZ, F. W., VINCENT, P. & RAVICH, R. B. M. (1971) Colony Stimulating Factor and Inhibitor Levels in Acute Granulocytic Leukemia. *Blood*, **38**, 143.

METCALF, D., PARKER, J., CHESTER, H. M. & KINCADE, P. W. (1974) Formation of Eosinophilic-like Granulocytic Colonies by Mouse Bone Marrow Cells *in vitro*. *J. cell. Physiol.*, **84**, 275.

METCALF, D., MACDONALD, H. F., ODARTCHENKO, N. & SORDAT, B. (1975) Growth of Mouse Megakaryocytic Colonies *in vitro*. *Proc. natn. Acad. Sci., U.S.A.*, **72**, 1744.

METCALF, D., WARNER, N. L., NOSSAL, G. J. V., MILLER, J. F. A. P. & SHORTMAN, K. (1975*a*) Growth of B-lymphocyte Colonies *in vitro* from Mouse Lymphoid Organs. *Nature, Lond.*, **255**, 630.

METCALF, D., NOSSAL, G. J. V., WARNER, N. L., MILLER, J. F. A. P., MANDEL, T. E., LAYTON, J. E., & GUTMAN, G. A. (1975*b*) Growth of B-lymphocyte Colonies *in vitro*. *J. exp. Med.*, **142**, 1534.

MOORE, M. A. S., SPITZER, G., WILLIAMS, N., METCALF, D. & BUCKLEY, J. (1974) Agar Culture

Studies in 127 Cases of Untreated Acute Leukemia. The Prognostic Value of Reclassification of Leukemia According to *in vitro* Growth Characteristics. *Blood*, **44**, 1.

SHERIDAN, J. W. & METCALF, D. (1974) Purification of Mouse Lung Conditioned Medium Colony Stimulating Factor (CSF). *Proc. Soc. exp. Biol. Med.*, **146**, 218.

STANLEY, E. R. & METCALF, D. (1969) Partial Purification and Some Properties of the Factor in Normal and Leukaemic Human Urine Stimulating Mouse Bone Marrow Colony Growth *in vitro*. *Aust. J. exp. Biol. med. Sci.*, **47**, 467.

STANLEY, E. R., HANSEN, G., WOODCOCK, J. & METCALF, D. (1975) Colony Stimulating Factor and the Regulation of Granulopoiesis and Macrophage Formation. *Fed. Proc.*, **34**, 2272.

STEPHENSON, J. R., AXELRAD, A. A., McLEOD, D. L. & SHREEVE, M. M. (1971) Induction of Colonies of Hemoglobin-synthesising Cells by Erythropoietin *in vitro*. *Proc. natn. Acad. Sci., U.S.A.*, **68**, 1542.